# Mangiferin and Cancer: Mechanisms of Action

**DOI:** 10.3390/nu8070396

**Published:** 2016-06-28

**Authors:** Fuchsia Gold-Smith, Alyssa Fernandez, Karen Bishop

**Affiliations:** 1 Auckland Cancer Society Research Center, Faculty of Medical and Health Sciences, University of Auckland, Private Bag 92019, Auckland 1142, New Zealand; fgol315@aucklanduni.ac.nz; 2 Faculty of Medical and Health Sciences, University of Auckland, Private Bag 92019, Auckland 1142, New Zealand; afer098@aucklanduni.ac.nz

**Keywords:** mangiferin, cancer, inflammation, NFκB, oxidative stress, cell cycle, combination therapy, nutraceuticals, bioavailability, hallmarks of cancer

## Abstract

Mangiferin, a bioactive compound derived primarily from Anacardiaceae and Gentianaceae families and found in mangoes and honeybush tea, has been extensively studied for its therapeutic properties. Mangiferin has shown promising chemotherapeutic and chemopreventative potential. This review focuses on the effect of mangiferin on: (1) inflammation, with respect to NFκB, PPARү and the immune system; (2) cell cycle, the MAPK pathway G_2_/M checkpoint; (3) proliferation and metastasis, and implications on β-catenin, MMPs, EMT, angiogenesis and tumour volume; (4) apoptosis, with a focus on Bax/Bcl ratios, intrinsic/extrinsic apoptotic pathways and telomerase activity; (5) oxidative stress, through Nrf2/ARE signalling, ROS elimination and catalase activity; and (6) efficacy of chemotherapeutic agents, such as oxaliplatin, etoposide and doxorubicin. In addition, the need to enhance the bioavailability and delivery of mangiferin are briefly addressed, as well as the potential for toxicity.

## 1. Introduction

Cancer has been identified as the leading cause of non-communicable disease mortality globally [1], and is responsible for significant morbidity and costs to healthcare systems. Cancer incidence and mortality has been increasing at a greater rate than population growth alone could account for. The International Agency for Research on Cancer (IARC) reported 14.1 million cases and over 8.2 million mortalities due to cancer in 2012 compared to 10 million cases and six million mortalities in 2000 [2] in a baseline population of 7.1 billion and 6.1 billion, respectively [3]. Much of this increase is due to rising cancer burden in less developed countries (LDCs), with 57% of new cases, and 65% of cancer related deaths occurring in LDCs [2]. When standardized by age, the total number of cases per 100,000 population is greater in more developed countries (MDCs) than LDCs (overall age standardized rate: 268 and 148 respectively) [4]. One exception to this pattern is infection-attributable cancers, which are responsible for 26% of the cancer burden in LDCs but only 8% in MDCs [5].

Cancer is less likely to be identified early or treated successfully in LDCs due to reduced access to screening tools and chemotherapeutic drugs. Previously, cancer has been regarded as a MDC disease. However, through the adoption of a more Westernised lifestyle, cancer incidence has been steadily increasing in LDCs. From the data published by Parkin et al., it can be seen that 40%–45% of cancers can be attributable to lifestyle factors such as diet, smoking status, alcohol consumption and lack of physical activity [6]. Some compounds naturally present in the diet, such as mangiferin in mangoes and honeybush tea, are thought to modulate risk of cancer and retard cancer progression.

Mangiferin (1,3,6,7-tetrahydroxyxanthone-C2-β-d glucoside) [7,8,9,10,11] is a polyphenol [8,11,12,13,14,15] found in many plant species, in particular, those from the Anacardiaceae [7,9,16,17,18,19,20] and Gentianaceae families [7,9,13,17,18,20]. For an extensive breakdown of plant sources of mangiferin and mangiferin content, see Matkowski et al. [21].

Mangiferin is not only present in everyday foods, but utilised in a number of natural medicines. In traditional medicine, different cultures have cultivated and processed mangiferin rich plants for the treatment of a range of illnesses including cardiovascular disease, diabetes, infection and cancer [22,23,24]. In India, Ayurvedic practitioners [22] have used *Salicia chinesis* (saptarangi) [21,25,26] and *Mangifera indica* (mango), which are two species that contain high levels of mangiferin. *Salicia chinesis* has been used for its hypo-lipidaemic, anti-diabetic, hepatoprotective and antioxidant properties. *Salicia chinesis* has now been over-exploited and research is being conducted into how this plant may be grown in a more sustainable way to meet demands [27]. *Mangifera indica* is used not only in Ayurvedic medicine but also used in Cuba [23], China [21,24] and throughout East Asia [21] for its anti-inflammatory, anti-viral, anti-diabetic and anti-cancer properties. *Mangifera indica*, a member of the Gentianaceae family, contains mangiferin [10,20,21,28,29,30] in its bark (18.33 g/kg dry weight [31]), leaves [15] (old leaves 36.9 g/kg and young leaves 58.12 g/kg dry weight [31]) and root along with the seed, pulp (0 to 2.65 mg/kg dry weight, depending on the variety [32]) and skin of the fruit [7,8,12,20,33,34,35] (4.94 g/kg dry weight [31]). However, the concentration of mangiferin in the pulp is unlikely to be sufficient to provide significant health benefits, and can vary greatly depending on variety and the maturity of the fruit [32]. Although somewhat lower than levels found in bark and leaves, the mangiferin in the skin [36] and seed/kernel [31], which are usually considered waste products, may provide a promising sustainable option for mangiferin extraction. To date, these mango by-products have been used to enhance the nutritional density of pasta, biscuits, muffins and pancakes [37,38,39,40]. Although the phenolic content of these food items increased 2.8–3.9 fold [37,39], the mangiferin content was not reported. However, in the results detailed in the sections hereafter, the concentrations used or administered varied from 12.5 to 100 µg/mL in in vitro studies [12] and approximately 100 mg/kg body weight in in vivo studies [7]. Clearly, the consumption of such quantities is not achievable by consuming fresh mango pulp, but maybe achievable by adding a leaf, bark, and/or seed extract as a supplement to food, or consuming as a liquid (if palatable).

In Cuba, aqueous extracts of *Mangifera indica* bark have become popular [7,12,41,42] for treatment of not only cancer but gastric and dermatological disorders, AIDS and asthma [43]. Stem bark extracts contain polyphenols, terpenoids, steroids, fatty acids and trace elements alongside mangiferin [21,23]. The natural medicine, Vimang^®^ [7,12,42], produced from aqueous extracts of *Mangifera indica,* contains ~20% mangiferin [23] and is available in tablets, creams and syrups. Vimang^®^ is registered as an “anti-inflammatory phytomedicine” by the Cuban Regulatory Health Authorities and is primarily used by those with multiple and different types of cancer. In China, mango leaves [21,24] and *Dobinea delavayi* (Baill.) leaves [44], which both contain mangiferin, are often used in traditional medicines. The greatest dietary source of mangiferin is Honeybush tea, popular in South Africa and obtained from *Cyclopia sp.* [21]. Honeybush tea leaves have been found to consist of up to 4% mangiferin by dry weight [21].

**Figure 1:**
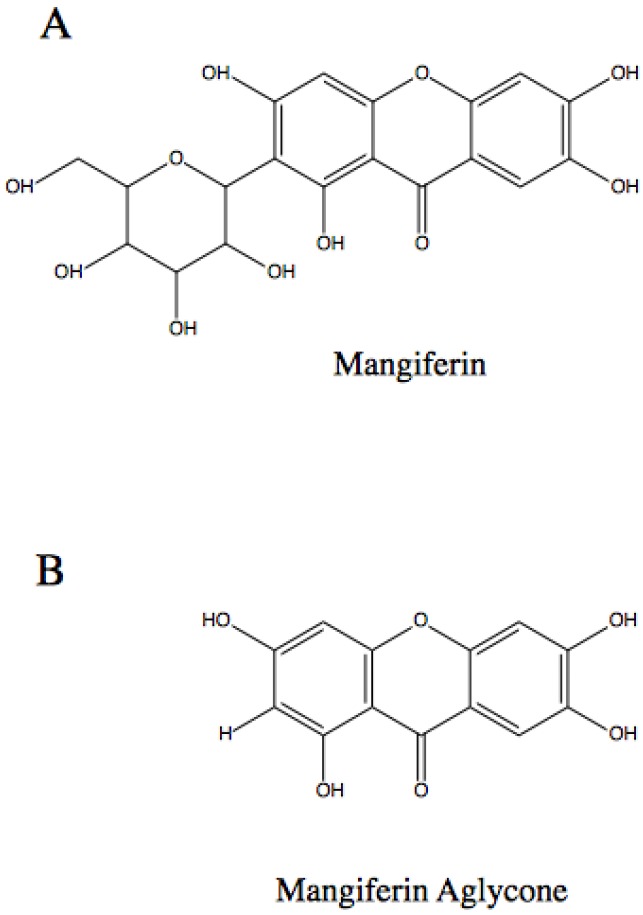
The molecular structure of: (**A**) mangiferin [45]; and (**B**) mangiferin aglycone [46].

Research into mangiferin has resulted in the identification of a similar compound, namely mangiferin aglycone or norathyriol, which appears to have greater biological activity in some instances. The compound mangiferin aglycone can be artificially synthesized, bypassing any sustainability concerns surrounding mangiferin. The structure of mangiferin and mangiferin aglycone are shown in Figure 1. Mangiferin aglycone has shown greater biological activity in some targets than mangiferin, possibly due to greater water solubility [28], and the former appears to reduce UV-induced skin cancer [8]. Further studies are required to elucidate the degree of similarity in action of mangiferin and mangiferin aglycone.

Evidence suggests that mangiferin could prove to be a useful, inexpensive compound to not only maintain and improve health in the worried well, but also to significantly improve the outlook for those with certain cancers (e.g., breast cancer [41]) and reduce the likelihood of developing cancer. This is of particular relevance to LDCs, where the more expensive chemotherapeutic drugs may be inaccessible, while mangiferin containing plants are abundant. In MDCs, the potential enhanced synergistic effect seen with major chemotherapeutic drugs may allow for lower dosages of drugs, thus reducing toxicity and providing greater selective toxicity to malignant cells, reducing the extent of side effects [47]. However, it is acknowledged that the quantity of fruit required in order to achieve clinically relevant levels of mangiferin may be unreasonably high. For this reason substitution of flour and sugar with mango processing by product [37,39] may prove an additional and useful method of increasing mangiferin intake.

The anti-cancer properties of mangiferin have been extensively studied over the past few decades. This review article seeks to consolidate the most recent research on the anti-neoplastic properties of mangiferin, with a focus on molecular pathways and uses of mangiferin, in conjunction with known chemotherapeutic agents, to aid further research on this topic.

## 2. Molecular Mechanisms of the Anti-Cancer Action of Mangiferin

Mangiferin acts through a myriad of mechanisms to exert anti-inflammatory [11,14,20,21,22,23,24,28,29,42,48], immunomodulatory [8,9,14,19,20,23,24,28,29,49], cell cycle arrest, anti-proliferative, anti-apoptotic [48], anti-oxidative [8,11,14,15,19,20,22,23,24,28,29,30,36,42,48,49,50,51], anti-genotoxic [30] and anti-viral [11,15,20,48] effects which cumulatively result in anti-tumour activity [9,11,15,19,20,21,23,24,29,41,50]. Mangiferin has demonstrated broad-spectrum efficacy against an array of different cancers in in vitro and in vivo studies [8,11,12,14,21]. To date, evidence suggests that the side effects of mangiferin vary from mild to non-existent [52]; however, there may be some variation according to source of mangiferin.

### 2.1. Inflammation

The chronic activation of inflammatory processes is widely regarded as an enabling characteristic towards the acquisition of cancer [53]. Approximately 20% of cancers are attributable to chronic inflammation [54], which may be induced by bacterial or viral infections, autoimmune disease, or constant exposure to irritants. Chronic inflammation can drive tumour growth by providing a favourable environment, rich in inflammatory mediators, to enhance cell growth and survival [53,55]. In addition, inflammation involves the production of reactive oxygen species (ROS), which can cause DNA damage, enhancing carcinogenic capabilities [56]. Mangiferin is thought to dampen down the inflammatory response primarily by interference with Nuclear Factor κ-light-chain-enhancer of activated B cells (NFκB) [34].

By reducing inflammation, mangiferin not only provides unfavourable conditions for cancer, but can provide anti-diabetic effects [11,15,19,21,23,24,28,29,50] and reduce risk of cardiovascular disease. Mangiferin also reduces serum glucose levels and lipid levels [8,14,30], further decreasing development and severity of diabetes and cardiovascular disease. Thus, while many medications used to treat these widespread non-communicable diseases may create adverse conditions in the body that may lead to other diseases, mangiferin provides broad spectrum benefits across a range of diseases such as cancers, cardiovascular disease and diabetes [26,33,35,39].

#### 2.1.1. Nuclear Factor κ-Light-Chain-Enhancer of Activated B Cells Activity

**Figure 2:**
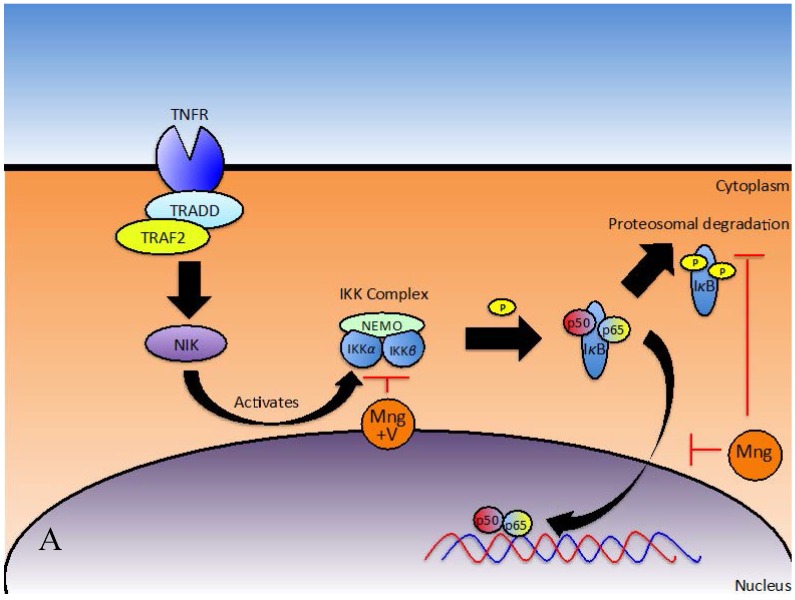

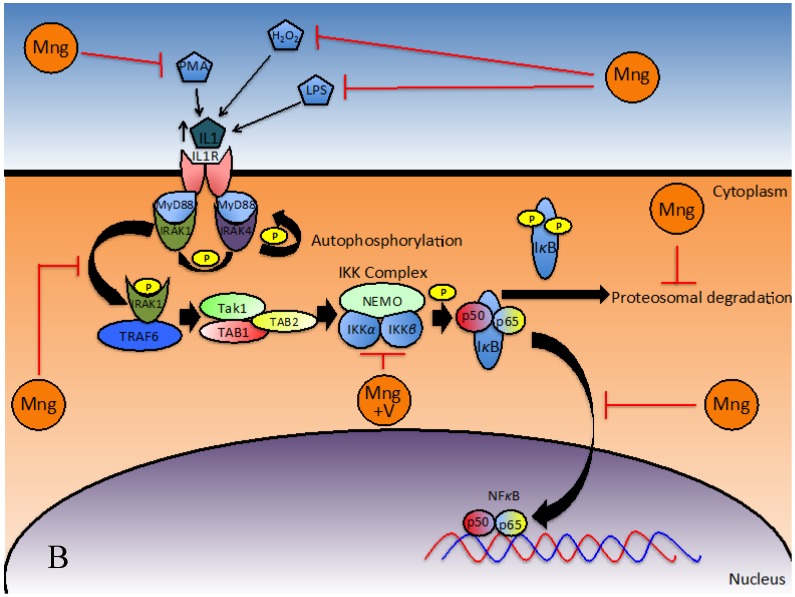
Inhibition of NFκB via the (**A**) classical and (**B**) alternative pathways by mangiferin and Vimang (adapted from [11,45,55]) (abbreviations: Mng, mangiferin; V, Vimang^®^).

The transcription factor NFκB regulates many important processes in inflammation, including the expression of pro-inflammatory cytokines, migration molecules, growth factors and other genes involved in proliferation and survival [34]. NFκB is up-regulated during inflammation. Under inflammatory conditions, ligands bind and activate Toll-like receptors (TLRs) and Interleukin-1 Receptors (IL-1R), triggering the Myeloid Differentiation Primary Response Gene 88 (Myd88) to recruit Interleukin-1 Receptor Activated Kinase 1 (IRAK1) to this receptor-signalling complex for phosphorylation [57,58]. Association of IRAK1 with Myd88 allows phosphorylation by IRAK4 and subsequent autophosphorylation. In its phosphorylated form, IRAK1 interacts with Tumour necrosis factor Receptor-Associated Factor 6 (TRAF6) to form a complex, which signals sequentially through Transforming growth factor beta-activated kinase 1/Transforming growth factor beta-activated kinase 1-binding protein 1 and 2 (TAK1/TAB1/TAB2), NFκB Essential Modulator/Inhibitor of NFκB Kinase subunit-β/Inhibitor of NFκB Kinase subunit-α (NEMO/IKK-β/IKK-α) and Inhibitor of κB (IκB)/p50/p65 complexes to ultimately activate NFκB [57]. Recent findings suggest mangiferin inhibits NFκB activation at various steps in the pathway (Figure 2A,B) [11,47]. NFκB can be activated via the classical or alternative pathways. The classical pathway is regulated by the IκB kinase complex and p50, while the alternative pathway is regulated by IKKα and p52 [59].

##### Initial Stimulus for NFκB Activation

When studied, it was found that mangiferin blocks Tumour Necrosis Factor (TNF) [8], lipopolysaccharide (LPS), peptidoglycan (PDG) [60], phorbol-12-myristate-13-acetate (PMA) [11] or hydrogen peroxide (H_2_O_2_) mediated NFκB activation by inhibiting ROS production [61]. This effect has been demonstrated in U-937 (lymphoma), HeLa (cervical cancer), MCF-7 (breast cancer) and IRB3 AN27 (human foetal neuronal) cell lines [11]. Jeong et al. [60] demonstrated that the inhibitory effect of mangiferin on NFκB expression when induced by LPS and PDG in peritoneal macrophages was elicited in part by inhibition of IRAK1 phosphorylation and consequently activation. In parallel, mangiferin impedes NFκB activation via inflammatory genes [11,48]. Inhibition of IRAK1 by mangiferin may reduce development of resistance to chemotherapeutic drugs. In particular, triple negative breast cancers have been associated with overexpression of IRAK1, and it is reported that inhibition of IRAK1, through the p38-MCL1 pathway, may reverse paclitaxel resistance [62]. Mangiferin, as a component of combination therapy, will be addressed in Section 4.

Subsequent studies have implicated mangiferin in suppressing the TNF signal transduction pathway [11,48], where under normal conditions, canonic interactions of TNF Receptor (TNFR) with Tumour Necrosis Factor Receptor type-1-Associated Death Domain protein (TRADD), TNFR-Associated Factor 2 (TRAF2) and NCK Interacting Kinase (NIK) along with subsequent phosphorylation and degradation of IκBα initiates NFκB activation (Figure 2A) [11]. To identify the site of action, U-937 cells were transfected with TNFR1, TRADD, TRAF2, NIK, IKK and p65 plasmids. Secreted Embryonic Alkaline Phosphatase (SEAP) was used as a reporter gene for NFκB and expression levels were monitored in treated and un-treated cells. Mangiferin inhibited TNFR1, TRADD, TRAF2, NIK and IKK induced SEAP expression but did not have a significant effect on p65 induced SEAP expression. Consequently, mangiferin must act downstream from IKK [11].

##### Signal Transduction to Activate NFκB

In a study carried out by García-Rivera et al. on estrogen negative MDA-MB231 breast cancer cells, the efficacy of Vimang^®^ (aqueous extract from *Mangifera indica*) was investigated and compared to treatment with either mangiferin only or gallic acid only (another bioactive present in Vimang^®^) [41]. At baseline, MDA-MB231 cells, which have a mutated p53 gene, demonstrate high NFκB activity [41]. When cells were pre-treated for 4 h with 200 µg/mL Vimang^®^ or 100 µg/mL of mangiferin, there was no change in IKKα expression, but reduced phosphorylation of IKKα and IKKβ was observed [41]. These proteins must be phosphorylated in order to transduce the signal and activate NFκB, thus mangiferin attenuated signal transduction. These authors also report that time taken for IκB phosphorylation and consequently degradation in response to TNF stimulation was doubled and time taken for IκB resynthesis was significantly reduced [41]. The action of mangiferin on IκB degradation has also been reported in a number of other studies [11,41,47,48,63]. Once IκBα is degraded, its inhibitory effect on the NFκB activation pathway is diminished [63] and thus NFκB can freely bind to DNA, allowing transcription and translation of the respective genes and proteins that it regulates [64]. Additionally, mangiferin and Vimang^®^ were found to reduce phosphorylation and translocation of p65 into the nucleus and impeded NFκB/DNA binding in response to TNF [41]. Other studies have also reported that mangiferin affects IκBα and p65 in this way [8,11,48]. García-Rivera et al. revealed that Vimang^®^, but not mangiferin alone was found to prevent parallel NFκB transactivation [41], emphasising the beneficial effects provided by other bioactive constituents of this aqueous extract.

It is clear that mangiferin is likely to attenuate NFκB expression in a multifaceted way, [34,47] with additional mechanisms yet to be elucidated.

##### Consequential Effects of NFκB Downregulation

NFκB is implicit in regulating expression of Cyclooxygenase-2 (COX-2), Intercellular Adhesion Molecule-1 (ICAM-1), B Cell Lymphoma-2 (bcl-2), Interleukin-6 (IL-6), Interleukin-8 (IL-8), C-X-C Chemokine Receptor type-4 (CXCR4), X linked Inhibitor of Apoptosis Protein (XIAP) and Vascular Endothelial Growth Factor (VEGF), which are all involved in inflammation, metastasis, cell survival and angiogenesis [11,29,42,48] (more on COX-2 below). As a downregulator of NFκB, mangiferin consequentially reduces expression of the genes listed above [41] and increases apoptosis [8].

IL-6 and IL-8 are both inflammatory cytokines that enhance cell proliferation. In MDA-MB231 cells, proliferation is conditional on autocrine synthesis of inflammatory cytokines and growth factors [41]. Vimang^®^ and mangiferin have each been found to down-regulate IL-6 and IL-8 production when stimulated by TNF [41], thus reducing the inflammatory response.

#### 2.1.2. Peroxisome Proliferator-Activated Receptor ү (PPARү)

PPARү is a nuclear receptor that also functions as a transcription factor, regulating expression of genes involved in cell differentiation and tumourigenesis [65]. Under normal circumstances, when the corresponding ligand binds to PPARү, transcriptional activation of COX-2 is suppressed through a number of mechanisms [66]. COX-2 is one of the key drivers of chronic inflammation through the production of prostaglandins leading to further activation of inflammatory processes [67], and thus COX-2 overexpression favours cancer progression [29]. PPARү also has a pleiotropic effect on blood glucose levels. PPARү agonists such as thiazolidinediones are widely used in management of diabetes and have a hypoglycaemic effect [65]. Hyperglycaemia is regarded as an emerging risk factor for cancer development [65]. Mangiferin, like thiazolidinedione may also act to reduce hyperglycaemia, benefiting diabetics and decreasing cancer risk.

Mangiferin increases mRNA expression of the PPARү gene [68] and thus decreases transcriptional activation of COX-2. This reduces inflammation and creates a less favourable environment for acquisition and proliferation of malignant cells. Mangiferin also impedes expression of COX-2 [41] via upregulation of TGF-β and downregulation of NFκB. Mangiferin may play a beneficial role in modulating PPARү and COX-2 regulation as evidenced by in vitro studies in MDA-MB231 breast cancer cells [43].

#### 2.1.3. Immune Response

Cancer cells can sometimes escape detection and avoid the immune system, which would otherwise destroy abnormal cells. Cancer cells not only express immune checkpoint proteins that dampen the immune response, but they may also release cytokines and growth factors that promote tumour cell proliferation and minimize apoptosis. By enhancing a patient’s immune response a better outcome can be achieved. In in vivo studies, mangiferin has been found to enhance the number and activity of immune cells [9,10].

Rajendran et al. found that in mice treated with benzo(a)pyrene (B(a)P) to induce lung cancer, dosing with mangiferin influenced the types of immune cells present and concentrations of various immunoglobulins [9]. Mangiferin treatment resulted in higher numbers of lymphocytes and neutrophils [9]. Mangiferin treatment of B(a)P mice increased levels of IgG and IgM immunoglobulins and decreased levels of IgA immunoglobulins, relative to animals only receiving B(a)P treatment [9]. In addition, mangiferin inhibited phagocytic capacity and nitric oxide production of macrophages when stimulated with LPS and IFNү [9]. Thus, with respect to the inflammatory response, less collateral damage is likely to occur. In a later study, it was found that in tumour bearing Swiss mice, mangiferin promoted cytotoxic behaviour of lymphocytes and macrophages against malignant cells, and thus the incidence of fibrosarcoma was reduced [7].

### 2.2. Cell Cycle

Maintenance of a normal cell cycle is essential for homeostasis. It allows cells to be replaced at the same rate as they are lost. Often in cancer, the length of the cell cycle is reduced, allowing aberrant proliferation of malignant cells.

Findings suggest that mangiferin influences the Mitogen Activated Protein Kinase (MAPK) pathway and progression from the G_2_/M checkpoint, thus maintaining a more normal cell cycle length, or cell cycle arrest at the appropriate checkpoint [8,13,52].

#### 2.2.1. Mitogen Activated Protein Kinase Pathway

The MAPK pathway is frequently implicated in tumourigenesis as it plays a role in processes such as cell proliferation, growth, differentiation, apoptosis and migration [69]. Mangiferin attenuates MAPK signalling [34] by inhibiting MAPKs p38, Extracellular signal-Regulated Kinase (ERK) and c Jun N-terminal Kinase phosphorylation [60]. Li et al. found that mangiferin aglycone, a metabolite of mangiferin, formed through deglycosylationin vivo, also inhibited ERK1/2 when phosphorylation was induced by UVB [70]. In this study, mangiferin aglycone was found to significantly reduce UV-induced skin cancers in mice, primarily through this interaction with ERK [70]. While further study is required, this suggests a beneficial effect against skin cancer.

#### 2.2.2. G_2_/M Checkpoint

Under normal conditions, cells with mutations are not able to undergo mitosis, as there are a number of checkpoints in the cycle that prevent mutated DNA from replicating [71]. Cancer cells must acquire characteristics that allow them to bypass these checkpoints in order to survive and proliferate [71].

**Figure 3:**
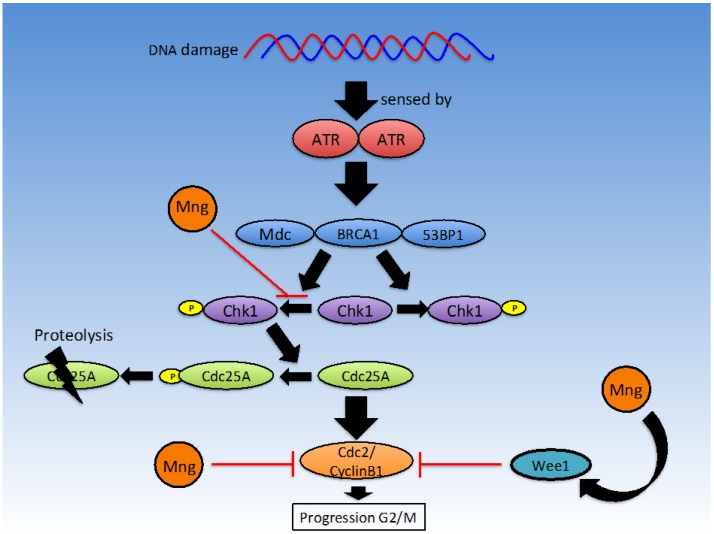
Mangiferin affects the molecular events leading to cell cycle G_2_/M phase arrest (Figure adapted from [71]).

The G_2_/M checkpoint occurs during the transition from G_2_ to mitotic entry. The G_2_ phase involves rapid growth of a cell as it prepares for mitosis. Cell progression from the G_2_/M checkpoint only occurs in the absence of DNA damage signals [72]. DNA damage can be sensed by Ataxia telangiectasia mutated protein (ATM) and Ataxia Telangiectasia and Rad3-related protein (ATR) which signal via Checkpoint kinase 1 (Chk1) and Checkpoint Kinase 2 (Chk2) to cause degradation of M-phase inducer phosphatase 1 (cdc25a), which results in inhibition of the Cyclin-Dependent Kinase 1 (CDK1)-cyclinB1 complex and thus cell cycle arrest [71,72] (see Figure 3). The cdc2-cyclinB1 complex is often overexpressed in malignant cells, enhancing entry into mitosis in eukaryotic cells. Malignant cells may acquire characteristics, which enable them to escape cell cycle arrest regardless of mutations. Chemotherapeutic agents such as etoposide target malignant cells at the G_2_/M checkpoint, thus when cell cycle progression is inhibited, the efficacy of etoposide at inducing apoptosis is increased. Mangiferin is thought to induce G_2_/M phase arrest [8], reducing proliferation of malignant cells and increasing efficacy of chemotherapeutic agents that target this phase.

Mangiferin has been shown to arrest cell cycle progression in a time dependent manner at the G_2_/M phase through suppression of the cdc2-cyclin B1 signalling pathway in MCF-7 cells [8]. This was observed through analysis of cell cycle distribution through flow cytometry, where a greater number of cells were found in the G_2_/M phase after incubation with mangiferin [13,52]. These findings are in keeping with results from the Peng et al. study in HL-60 cells [8]. Peng et al. [52] also found that in HL-60 leukaemia cells, gene expression of Chk1, cdc25 and Wee1 was elevated when exposed to low concentrations of mangiferin, but at higher concentrations, Chk1 and cdc25 gene expression was reduced at the mRNA level. Mangiferin has been shown to significantly inhibit phosphorylation of ATR, Chk1 and other proteins with anti-proliferative properties such as Wee1, Akt and Erk1/2, while increasing phosphorylation of cdc2 and cyclinB1 [52]. Lv et al. used a Western blot assay to identify a reduction in cdc2 (cdk1) and cyclinB1 [8] protein levels in response to treatment with mangiferin. Findings suggest that inhibition of the ATR-Chk1 stress response DNA damage pathway by mangiferin is responsible for cell cycle arrest.

While G_2_/M phase arrest has been identified in response to mangiferin treatment in a number of cancer cell lines (MCF-7, HL-60, BEL-7404 and CNE2) [16,18,35,52,73], further study is required to determine dosages of mangiferin required to elicit an effect. In addition to G_2_/M phase arrest, Lv et al. also suggest that mangiferin may induce G_0_/G_1_ cell cycle arrest in MCF7 cells [8].

### 2.3. Proliferation/Metastasis

Under normal circumstances, the rate of cell replication and cell death is matched to maintain homeostasis. In cancer cells, the mediators of these processes may be deregulated, allowing cells to proliferate continuously, exceeding rates of cell death. Cancer cells may develop a more motile phenotype, due to deregulation of cell adhesion pathways. Loss of adhesion allows cells to escape their site of origin and spread to other sites, causing secondary malignancies.

Mangiferin is thought to reduce cell proliferation [16] through modulation of β-catenin and consequently metalloproteinase-7 (MMP-7), MMP-9, and EMT (epithelial to mesenchymal transition) [14]. Through NFκB, mangiferin may influence VEGF-A transcription to modulate angiogenesis. Additionally, in in vivo experiments, mangiferin has shown efficacy at reducing tumour volume in mice [14].

In a variety of breast cancer cell lines, mangiferin has been implicated in reduced cell proliferation (MDA-MB-231, BT-549, MCF-7 and T47D) [8,14] and reduced metastasis (MDA-MB-231 and BT-549) in a dose-dependent manner [14]. In HL-60 cells, Li et al. reinforced that mangiferin reduced proliferation [14]. In contrast, Wilkinson et al. found that mangiferin did not suppress proliferation in MCF-7 cells, while mangiferin aglycone did [74]. This may be a result of differential activation of estrogen receptors [74]. Kim et al. also reported no significant effect on proliferation when HeLa cells were treated with 25–200 µM of mangiferin [36] and Garcia-Rivera et al. found no significant inhibition of proliferation in MDA-MB231 cells when treated with mangiferin, but proliferation was inhibited by Vimang^®^ [41]. Thus, further evidence is required to ascertain an effect.

#### 2.3.1. Glycogen Synthase Kinase-3β/β-Catenin

In cancer, aberrant activation of β-catenin is often observed [14]. High levels of expression of β-catenin are associated with proliferation and metastasis. Glycogen synthase kinase -3β (GSK-3β) is capable of phosphorylating and degrading β-catenin [14]. GSK-3β may be inhibited by a number of signals. Mangiferin is hypothesised to suppress the β-catenin pathway [14].

Using a Western blot assay to analyse protein expression in breast cancer cell lines, mangiferin was found to down-regulate β-catenin and decrease levels of inactive GSK-3β, indicating suppression of the β-catenin pathway, which in turn down-regulates MMP-7, MMP-9 and snail expression [14]. Snail can be used as an epithelial/mesenchymal phenotye indicator [14], thus lower levels of snail, which are seen on exposure to mangiferin, favour a more epithelial, less mobile phenotype, while higher expression of snail would indicate a more motile phenotype, allowing malignant cells to metastasise.

#### 2.3.2. Matrix Metalloproteinases

Activation of matrix MMPs is a crucial step towards metastasis as these enzymes facilitate cell escape from the initial site of the malignancy, through degradation of the extracellular matrix. As above, mangiferin has been linked to downregulation of NFκB, which in turn influences downstream expression of MMPs [64,75].

In breast cancer, the matrix metalloproteinases MMP-2, -7 and -9 are often up-regulated [14]. Li et al. have demonstrated through a Western blot assay that of these three enzymes, MMP-2 was not significantly affected while MMP-7 and MMP-9 were down-regulated by mangiferin [14]. MMP-7 and -9 strongly promote cancer progression by allowing malignant cells to metastasise [76]. In LNCaP prostate cancer cells, activation of NFκB by TNF-α increases levels of MMP-9 mRNA and protein present in the cell [75]. Mangiferin is capable of attenuating this effect, ultimately reducing metastasis [75]. In addition to this pathway of MMP-9 activation, Xiao et al. (2015) discovered that mangiferin stimulates miR-15b expression, which in turn down-regulates MMP-9 expression in U87 glioma cells [16], thus reducing the capability of malignant cells to escape the extracellular matrix and metastasise. In the study by Jung et al., mangiferin prevented PMA induced MMP-9 expression without influencing other MMP expression in human astroglioma cell lines: U87MG, U373MG and CRT-MG [19]. MMP-1, -2, -3 and -14 expressions were not influenced by mangiferin [19]. Mangiferin is thought to act by suppressing NFκB and AP-1 binding to the promoter region of MMP-9 and prevents phosphorylation of Akt and MAP kinases (see above section) induced by PMA [19]. Jung et al. also suggest that mangiferin acts on MMP-9 suppressors, *Tissue Inhibitor of Metalloproteinase -1* and *-2* (*TIMP-1* and *-2*). *TIMP-1* and *TIMP-2* mRNA levels were enhanced by the presence of mangiferin, implying another favourable quality of mangiferin [19]. Jung et al. suggest that mangiferin, through these mechanisms, may reduce glioma invasiveness [19]. Overall, published studies indicate that mangiferin may play an important role in reducing expression of MMP-9, limiting cancer invasiveness [16,19].

#### 2.3.3. Epithelial to Mesenchymal Transition

EMT involves the loss of adherence and gain of a motile phenotype and resistance to apoptosis, which may allow motile cancer cells to migrate from their site of origin and survive, causing secondary metastases [53]. β-catenin signalling may also play a role in EMT [14]. Mangiferin appears to enhance epithelial characteristics in breast cancer cell lines and thus help protect against metastasis [14].

Li et al. [14] investigated the effect of mangiferin on EMT through analysis of two mesenchymal-like breast cancer cell lines (MDA-MB-231 and BT-549). Mesenchymal characteristics were reduced upon treatment with mangiferin, whereby cells obtained a more epithelial-like morphology. Associated with these physical observations, increased expression of the epithelial phenotype marker, E-cadherin, and decreased expression of mesenchymal phenotype markers, vimentin, snail and slug were seen [14]. In MDA-MB-231 xenograft mice treated with mangiferin, Western blot analysis revealed the same shift in expression in epithelial and mesenchymal markers with lower expression of active β-catenin, MMP-7, MMP-9 and vimentin (mesenchymal markers) and higher expression of E-cadherin (an epithelial marker) [14], reinforcing the in vitro results. While these results are promising in breast cancer cells, investigation in a more diverse range of cell lines is required to determine if these findings may be applicable to a broader range of breast cancer cell lines as well as other cancer cell lines.

#### 2.3.4. Angiogenesis

Sustained angiogenesis is widely regarded as an enabling characteristic of cancer, as tumours are unable to survive beyond a certain size without their own blood supply [53]. Angiogenic tumours are able to grow and proliferate using nutrients and oxygen from their own blood supply. The VEGF-A protein is known to stimulate angiogenesis [53]. Both mangiferin and Vimang^®^ extracts have demonstrated inhibitory effects on TNF-induced transcription of VEGF-A in MDA-MB231 cells [41]. However, this experiment was carried out over a short time period. Further investigation over longer time periods and evidence from in vivo/ex vivo studies are required to further determine the effect of mangiferin on angiogenesis.

#### 2.3.5. Tumour Volume

Duringin vivo experiments in mice, mangiferin has been found to reduce tumour volume. In C57BL/6J mice inoculated with MCF-7 cells on the neck, a reduction of 89.4% in tumour volume relative to control was seen when mice were medicated with 100 mg/kg of mangiferin. This value was closely comparable to the results obtained from cisplatin treatment (91.5%), an established chemotherapeutic drug [8]. In a similar experiment, the lifespan of these mice was extended at dosages from 10 mg/kg mangiferin and above and 60% of mice survived until the end of the assay period [8], while in the no treatment group, there were no mice surviving after day 40 following MCF-7 inoculation. A high dosage of mangiferin (100 mg/kg) extended lifespan to the same degree as cisplatin, with no significant difference (*p* < 0.05) being observed between these treatments [8]. Dose dependency was observed [8].

These results show that mangiferin can act as a potent chemotherapeutic agent in mice and thus further investigation into mangiferin-based products could benefit treatment of cancer in humans.

### 2.4. Apoptosis

**Figure 4:**
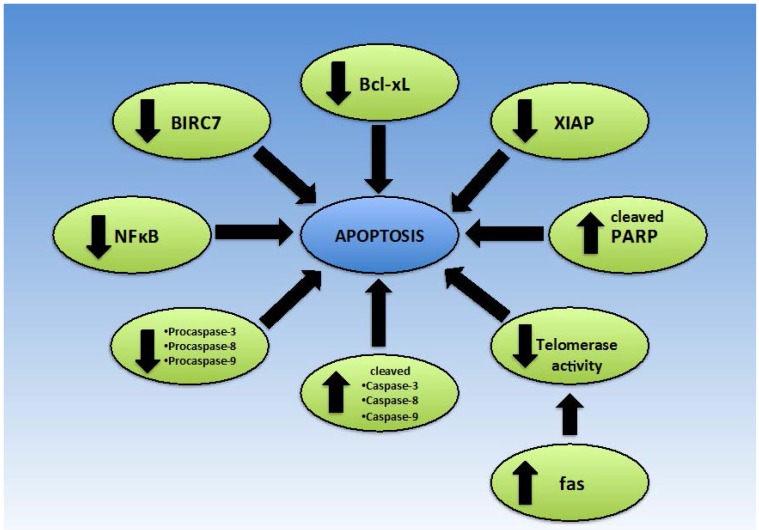
Effect of Mangiferin on proteins implicated in apoptosis.

In order to survive and proliferate, cancer cells must be able to evade apoptosis, despite carrying malignant characteristics [53]. Under normal circumstances, either the intrinsic pathway via the mitochondria, or the extrinsic pathway involving death receptors, can induce apoptosis. The intrinsic pathway generally involves increased permeability of the mitochondrial membrane and the release of cytochrome C to activate initiator procaspase-9, while the extrinsic pathway involves Fas Associated Death Domain (FADD) and procaspase-8 [36] (Figure 4). Apoptosis is the preferred pathway of cell death, as necrotic cell death may induce inflammatory changes due to the release of immune-stimulatory molecules. In order to eradicate cancer, many chemotherapeutic agents seek to induce apoptosis in malignant cells. From the peer reviewed literature, it can be concluded that mangiferin has promising apoptosis inducing properties in a number of cell lines and is involved in regulating apoptosis via multiple targets [8,14,36].

In 2013, two studies were published that demonstrated a dose dependent increase in apoptosis in response to increasing mangiferin concentration in MDAMB-231, BT-549, MCF7 and T47D breast cancer cell lines [8,14]. Kim et al. reported similar findings in HeLa cells in response to treatment with ethanolic extracts of mango skin or flesh [36]. There are a number of suggested mechanisms by which an increase in apoptosis in these cancer cells may be potentiated. As discussed earlier, mangiferin down-regulates the transcription factor NFκB. It is hypothesized that this dampening of NFκB activity is likely to be responsible for increased apoptosis in HL-60 acute myeloid leukaemia (AML) cells, MCF7 cells and HeLa cells [8,13,35,77].

#### 2.4.1. Mangiferin and Hesperidin in *Cyclopia* Sp. Extracts

Bartoszewski et al. showed in HeLa cells that treatment with *Cyclopia sp.* tea extracts, which are high in mangiferin and hesperidin, caused up-regulation of TRADD and TNFR superfamily member 25 (TRAMP), which are involved in signalling of the extrinsic apoptotic pathway [77]. However, when compared to mangiferin only and hesperidin only, it would appear that hesperidin is a more potent activator of apoptosis in HeLa cells than mangiferin [77]. Regardless, mangiferin did enhance the activity of hesperidin, even when added in low concentrations. Mangiferin itself caused down-regulation of Baculoviral IAP Repeat Containing 7 (BIRC7), which sensitizes cells to death by the extrinsic apoptotic pathway [77].

#### 2.4.2. Bax/Bcl-2

The Bcl-2 protein acts to block programmed cell death while the Bcl-2 associated X protein (Bax) protein favours apoptosis. When the ratio of Bax:Bcl-2 is increased, a cell’s sensitivity to apoptosis is increased [78], and consequently malignant cells are less likely to survive. Current literature suggests that the effect of mangiferin on the Bax:Bcl-2 ratio is dependent on cell type, dosage and perhaps the form of mangiferin used [35,79].

Pan et al. found that when CNE2 nasopharyngeal carcinoma cells were treated with mangiferin, the mRNA and protein expression levels of Bcl-2 were consistently down-regulated while Bax was up-regulated [35]. As a consequence, these cells were primed for apoptosis. Bcl-2 was also down-regulated upon treatment with an ethanolic extract of mango skins, which contained mangiferin, mangiferin gallate and isomangiferin gallate [36]. This ultimately resulted in activation of caspase-3, -6, -8 and -9 alongside poly (ADP-ribose) polymerase (PARP) protein [36], favouring cell death. However, Klavitha et al. [22] have found that the reverse applies in the context of excitotoxicity in neurons, whereby mangiferin blocks upregulation of Bax, thus attenuating cell death, making it a promising compound for further research with regard to Parkinson’s disease [22]. Furthermore, Bartoszewski et al. demonstrated that on analysis of green fermented *Cyclopia* sp. extracts (in which the primary compounds were mangiferin and hesperidin), there were no significant changes in Bax/Bcl2 mRNA levels or protein levels [77], although Bartoszewski et al. acknowledge that the most likely cause of this disparate finding was low dosage.

In addition to the Bax/Bcl2 ratio, Zhang et al. and Pan et al. reported that apoptosis could be triggered by mangiferin in HL-60 cells due to changes in levels of similar proteins [13,35]. HL-60 cells responded to mangiferin by decreasing levels of Bcl-extra large (Bcl-xL) and XIAP [13,35], resulting in increased apoptosis.

Further experimentation in a wider range of cell lines is required to elucidate what dosage of mangiferin is likely to provide an effect.

#### 2.4.3. Intrinsic/Extrinsic Apoptotic Pathway

To identify whether mangiferin was acting on the intrinsic or extrinsic apoptotic pathway, Kim et al. performed a Western blot experiment to assess expression levels of proteins involved in either the intrinsic pathway, extrinsic pathway or both pathways [36]. Results indicated that there was slightly lower expression levels of BH3 interacting domain (Bid), pro-caspase-3 and pro-caspase-8, but increased expression of cleaved, active forms of PARP, caspase-7 and caspase-9 [36], when HeLa cells were treated with an ethanolic extract of mango peel. Consequently, it is likely that the ethanolic extracts of mango pulp and skin influenced both apoptotic pathways, which is crucial for effective apoptosis. Lv et al. further strengthened the evidence for the role of mangiferin in the intrinsic apoptotic pathway by considering cytochrome C [8]. They found that when MCF-7 cells were treated with mangiferin, cytochrome C concentration in the mitochondria was reduced, while a corresponding increase in cytochrome C concentration was observed in the cytosol. This indicates that cytochrome C was released from the mitochondria in response to mangiferin treatment and thus apoptosis may be induced via the mitochondrial pathway [8]. In addition to these findings, increased expression of caspase-3, -8 and -9, and decreased expression of procaspase-3, -8 and -9 expression was noted, suggesting activation of both intrinsic and extrinsic apoptotic pathways [8]. Based on results from their study, du Plessis-Stoman et al. have suggested that mangiferin may favour apoptotic cell death over necrotic cell death, which has potential to reduce inflammation [48].

#### 2.4.4. Telomerase

Aside from the study of various pathways of apoptosis, in the literature it is reported that mangiferin can inhibit telomerase activity in K562 human leukaemia cells with dose- and time- dependent behaviour [8,35,80], promoting apoptosis. It has been suggested that this may be due to increased *fas* gene expression and protein levels of fas [8]. Enhanced telomerase activity is found in a variety of cancers and is permissive and required for sustained growth of late cancers. Almost all cancers exhibit some form of telomerase reactivation [81]. By reducing telomerase activity, mangiferin can be used to reduce the progression of existing cancers and create an environment in which malignant cells are unlikely to survive.

Mangiferin has demonstrated pro-apoptotic activity in a number of cancer cell lines including K562 leukaemia, MCF-7 breast cancer and CNE2 nasopharyngeal cells [8,35].

### 2.5. Oxidative Stress

**Figure 5:**
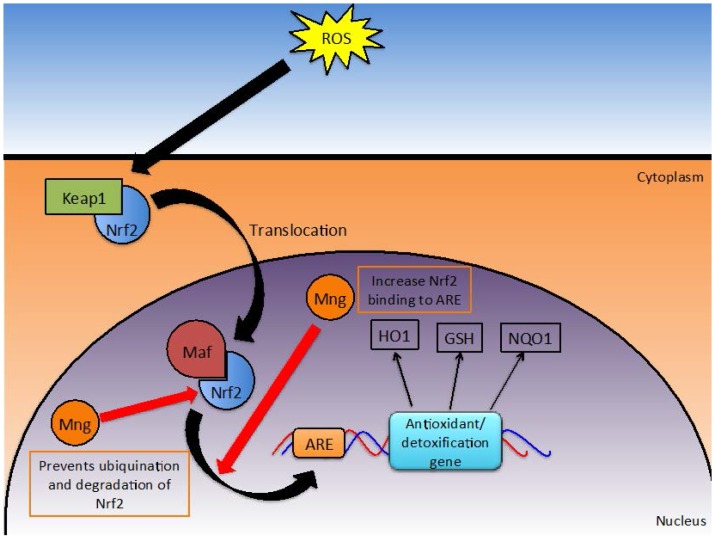
Effect of Mangiferin on the Nrf2/ARE Detoxification Pathway.

Oxidative stress occurs when the burden of ROS is not balanced by antioxidants and detoxification systems. The presence of these excess reactive species can result in cellular damage, particularly to DNA, lipids and proteins. Over time, oxidative stress increases the risk of developing cancer and may exacerbate inflammation. Mangiferin is thought to play a role in: (1) modulating the Nrf2/antioxidant response element (ARE) detoxification pathway (Figure 5); (2) directly detoxifying reactive species; and (3) activating detoxification enzymes such as catalase.

#### 2.5.1. Nrf2/ARE Detoxification Pathway

Under normal conditions, Nrf2 gene transcription is inhibited by Kelch-like ECH-associated protein-1 (KEAP-1). However, oxidative stress, dietary components and synthetic chemicals can induce Nrf2 transcription [18]. Consequently, Nrf2 protein can accumulate in the nucleus where it forms heterodimers with musculoaponeurotic fibrosarcoma (maf) protein. This heterodimer signals through the ARE to initiate transcription of a number of phase II detoxification enzymes [17], such as NAD(P)H: quinine reductases (NQO1), glutathione S-transferase (GSH) and heme oxygenase (HO-1) [18]. HO-1, when activated, can translocate into the nucleus to further activate transcription factors relevant to the stimulus [82]. Ultimately, this pathway provides activation of detoxification enzymes when oxidative stresses are presented. Mangiferin manipulates this pathway in such a way that the survival of healthy cells but not malignant cells is enhanced. Mangiferin modulated this Nrf2/ARE signaling pathway at multiple steps [13,17,18].

While mangiferin does not directly influence Nrf2 transcription rates, Zhao et al. have demonstrated that the half-life of Nrf2 is increased due to impaired ubiquitination and thus degradation of the protein [18], which results in higher levels of the protein being present within the cell. Zhang et al. also reported similar findings in human umbilical cord mononuclear blood cells, where mangiferin increased the quantity of Nrf2 accumulating in the nucleus in a time dependent manner [17]. Protein quantity was assessed by microscopy and verified by Western blotting [17]. Additionally, mangiferin increased the binding of Nrf2 to ARE which in turn was shown to increase downstream production of NQO1 (a prominent antioxidant enzyme) when assessed in a Western blot assay [13,17].

Nrf-ARE signaling can provide protection against agents that are chemotherapeutic to normal cells [13] (more on synergistic effects of mangiferin and chemotheraputics later). Similarly, overexpression of Nrf2 in cancer cells can promote resistance to therapy, through up-regulation of antiapoptotic bcl-xL. Mangiferin seems able to differentiate between malignant cells and healthy cells, promoting Nrf2 activation in healthy cells (human umbilical cord mononuclear blood cells) but not cancerous cells (HL-60). Thus, survival is aided in healthy cells by enhanced efficiency of the Nrf2/ARE detoxification pathway while the development of resistance to chemotherapeutics is not permitted in malignant cells [13]. To date, mangiferin is the only known Nrf2 activator that does not confer protection to malignant cells against chemotherapeutic agents [13], making it a promising agent for cancer therapy.

Downstream effects in the Nrf2/ARE detoxification pathway have been further studied upon treatment with Vimang^®^. Treatment of MDA-MB231 breast cancer cells with 200–400 µL/mL of Vimang^®^ was found to significantly increase HO-1 transcription. However, when treated with mangiferin alone, there was no significant increase in HO-1 transcription [41]. From this result, one may deduce that the Nrf2/ARE detoxification pathway may not have been activated by mangiferin, as was reported earlier in the HL-60 cancer cell line. It is possible that an alternative bioactive, found in Vimang^®^ may be responsible for the up-regulation of HO-1 transcription in the MDA-MB231 breast cancer cells. Overall, results would suggest that mangiferin may provide some benefit through activation of the Nrf-ARE detoxification pathway.

#### 2.5.2. Elimination of Reactive Species

Reactive species must be eliminated promptly to avoid damage to important biological molecules. This may be done directly by antioxidant species, or by inducing and up-regulating detoxification pathways. Mangiferin is an established antioxidant that is able to neutralize a range of reactive species and influence expression and activity of key detoxification enzymes. By performing these actions, oxidative stress and inflammation are reduced.

Mangiferin is able to directly protect against hydroxyl [28], 2,2-diphenyl-1-picrylhydrazyl (DPPH), superoxide, hydrogen peroxide [51], and peroxynitrite free radicals, lipid peroxides [9,21], hypochlorus acid [28] and heavy metal induced reactive oxygen species [15]. Findings from numerous studies can be used to reinforce the notion that mangiferin has greater or comparative antioxidative capacity to other known antioxidants, such as quercetin, baicalein, catechins, phenylpropanoic acids [21], vitamin C, vitamin E and β-carotene [28]. Alongside its antioxidative potential, mangiferin influences ROS production through modulating Fenton-type reactions. Fenton-type reactions usually involve the production of a hydroxyl radical and the oxidation of Fe^2+^ to Fe^3+^. In the presence of mangiferin, Fenton-type reactions are inhibited by chelating Fe^2+^ ions, reducing production of subsequent ROS [15,29,51]. Additionally, Duang et al. have suggested that mangiferin protects against lipid peroxidation [29]. This protection may in part be responsible for reduced DNA damage and amelioration of cytotoxic action seen in response to ionising radiation in healthy cells [9,83].

Both in vitro and in vivo evidence suggests that mangiferin up-regulates expression of various detoxifying enzymes, resulting in enhanced clearance of ROS. In N2A neuroblastoma cells, Kavintha et al. implicated mangiferin in reducing oxidative stress by providing protection against 1-methyl-4-phenylpyridine (MPP^+^) induced cytotoxicity, due to its capability to restore glutathione action and reduce expression of superoxide (SOD) and catalase [22]. In addition to these findings, Matkowski et al. also reported that mangiferin influenced SOD, catalase and glutathione peroxidase in such a way that it halts ROS centred apoptotic pathways through dampening endogenous ROS production [21]. Sarker et al. demonstrated the relationship between mangiferin and glutathione levels by showing that mangiferin increased levels of GSH more than 2× the amount observed on treatment with other anti-oxidants [11]. It has been suggested that mangiferin increases GSH levels by up-regulation of ү-Glutamylcysteine Synthetase (ү-GCS), the enzyme controlling the rate limiting step of GSH synthesis [11]. In vivo studies demonstrate a similar pattern of increased detoxification enzyme activity. In experiments using B(a)P-treated mice, B(a)P attenuated SOD and catalase (see below for more on catalase) activity in lymphocytes, polymorphonuclear cells and macrophages [9]. However, mangiferin co-administration provided a protective effect against these events. Rajendran et al. also found that mangiferin reduced the production of H_2_O_2_ in B(a)P treated animals [9]. In animals with lung cancer, enhanced activity of glutathione transferase [48], quinine reductase and uridine 5’-diphosphate-glucuronosyl transferase activity has been demonstrated upon treatment with mangiferin [8]. These events each contribute to a reduction in oxidative stress through increased capacity to deal with assault from reactive species. Sarker et al. further suggest that the ability of mangiferin to reduce oxidative stress may also be linked to NFκB down-regulating capabilities, which reduces TNF-induced reactive oxygen intermediate generation [11].

#### 2.5.3. Catalase

Catalase is a detoxification enzyme present in most organisms exposed to oxygen that converts H_2_O_2_ into water and oxygen. H_2_O_2_ can cause oxidative damage if not rapidly converted into less toxic species. Mangiferin may directly increase the efficiency of the catalase enzyme by interacting directly with the enzyme, thus reducing oxidative damage that can be done prior to detoxification of H_2_O_2_ [61]. Increased activity of catalase may modulate downstream signalling pathways that favour an environment that does not promote cancer development and survival. However, not all published findings are consistent with the notion that mangiferin increases catalase activity [11,61].

In silico docking studies using AutoDock and PyMol predict that mangiferin has the capacity to bind to the active site of catalase, but not other oxidase enzymes [61]. The binding of mangiferin to catalase enhanced the activity of catalase by 44% during the in vitro studies conducted by Sahoo et al. [61]. An earlier study by Sarkar et al. reported disparate findings, where mangiferin caused a 0%–23% increase in activity when compared to untreated cells, and did not influence the quantity of enzyme present [11]. In both experiments, U-937 cells were treated alongside other cell lines with 10 µg/mL of mangiferin for 3 h [11].

To further elucidate the effect of mangiferin on catalase activity, Sahoo et al. conducted fluorescent spectrophotometry experiments on catalase in the unbound state (peak at 330 nm, excitation wavelength 280 nm) and subsequently, increasing concentrations of mangiferin were added [61]. As the concentration of mangiferin was increased, the peak at 330 nm decreased in magnitude, suggesting interaction with mangiferin. When the binding constant was calculated (3.1 × 10^−7^ M^−1^), this indicated a strong binding affinity between catalase and mangiferin [61]. Mangiferin also proved capable of overriding aminotriazole (ATZ) inhibition of catalase in lipid peroxidation assays [61]. Sahoo et al. further demonstrated that direct quenching of H_2_O_2_ by mangiferin was not significant, implying that the entire 44% difference found may be attributable to enhanced activity of the catalase enzyme [61].

It has been suggested that increased catalase activity may dampen excessive activation of MAPK/AKT, which is commonly found in malignant cells [61] (See above for MAKP/AKT). Sarker et al. suggested that high expression of catalase would reduce NFκB levels [11]. However, evidence does not support any change in catalase expression, only in the efficiency of this enzyme [11,61]. Increased catalase activity could reduce oxidative stress and inflammation, thus favouring a chemopreventative environment.

### 2.6. DNA Damage

DNA damage facilitates mutations in the genetic material of a cell. Mutation is required to initiate the development of cancer and also expedites the acquisition of characteristics required for a malignant cell to survive. Thus, a higher susceptibility to DNA damage results in a higher incidence of mutation and the development of cancer [84]. The role of mangiferin with regard to DNA damage is controversial.

Studies have reported that mangiferin is capable of protecting not only DNA [42] but also deoxyribose, phospholipids, polyunsaturated fatty acids and proteins [21]. However, Rodeiro et al. [12] found that when aqueous extracts from *Mangifera indica* bark were applied to lymphocytes and lymphoblastic cells, DNA damage was induced. When this effect was further investigated with the compound mangiferin alone, there was a reduction in DNA damage, thus there is likely to be an alternative compound in the extract that is inducing DNA damage [12]. In addition, Rodeiro et al. found that when DNA damage was induced by γ-radiation, the aqueous extract was protective against DNA damage [12].

#### Radiation Damage

Ionising radiation has been shown to induce DNA damage. In patients undergoing radiotherapy, many healthy cells acquire collateral damage. Mangiferin and mangiferin aglycone have demonstrated protective effects against radiation damage during in vitro studies [28].

Lei et al. demonstrated that pre-treatment of human intestinal epithelial cells with mangiferin aglycone reduced the percentage of cells with double strand breakages in their DNA by 47% when treated with ionizing radiation [28]. This was more effective than the 40% reduction seen following mangiferin pre-treatment. Currently, there are few radioprotective agents, and these agents tend to be associated with high levels of toxicity [28]. Mangiferin may provide some protection to cancer patients undergoing chemotherapy as well as improve efficiency of anti-cancer treatments.

## 3. Synergistic Effects

The use of many chemotherapeutic agents induces a range of side effects, which can cause serious illness. Mangiferin shows potential to reduce or negate these side effects by selectively targeting malignant cells for cell death and enhancing survival of healthy cells. Mangiferin may potentiate cell death by existing drugs through modulation of NFκB activity [11] and causing cell cycle arrest in malignant cells at the G_2_/M checkpoint, leaving cells susceptible to apoptosis induced by chemotherapeutic agents such as etoposide [13]. Through NFκB inhibition, mangiferin is likely to reduce resistance to chemotherapeutic agents in cancer cells [13,48]. Studies using pro-apoptotic agents such as oxaliplatin, etoposide, doxorubicin and paclitaxel have documented additional beneficial effects when co-administered with mangiferin (Table 1).

### 3.1. Pro-Apoptotic Agents

While mangiferin (at a concentration of 10 µg/mL) does not trigger apoptotic cell death itself [11], it may enhance action of chemotherapeutic pro-apoptotic agents. Sarker et al. [11] demonstrated that this was due to down-regulation of NFκB by transfecting U-937 cells with an IκBα-double negative construct, blocking NFκB activation and also transfecting with a p65 construct and observing cell death after 36 h by MTT assay, using the Live/Dead cell assay. In IκBα-double negative transfected cells, cell death increased by 12% and increased cell death with TNF from 42% to 53%. Cell death in the presence of mangiferin was increased a further 4%. In p65 overexpressing cells, cell death was not observed in response to treatment with TNF or TNF and mangiferin. By considering SEAP as a reporter gene, IκBα-double negative cells were shown to down-regulate NFκB and p65 overexpressing cells up-regulated NFκB. Thus, it was found that down-regulation of NFκB primes cells for cytotoxic agents [11].

Sarkar et al. reported that the activity of the pro-apoptotic agents cisplatin, vincristine, doxorubicin, etoposide, Adriamycin and AraC was enhanced significantly by co-administration of mangiferin in U-937 cells [11]. Unlike other antioxidants, mangiferin was not found to be toxic to the cells, as it only enhanced cell death when exposed to TNF [11].

Oxidative damage induced by chemotherapeutic drugs correlates with the development of secondary malignancies such as acute myeloid leukaemia (AML). Mangiferin reduces oxidative stress induced by these agents and thus reduces likelihood of developing secondary malignancies [13].

By enhancing apoptotic activity against malignant cells upon treatment with chemotherapeutic agents, lower dosages may be required when co-administered with mangiferin, which may reduce the side effects associated with toxicity.

#### 3.1.1. Oxaliplatin

Oxaliplatin is a platinum-based anti-neoplastic agent used for the treatment of colon or rectal cancer once metastasised. It is often given in conjunction with other chemotherapeutic agents. Common side effects, occurring in >30% of patients, include nausea, vomiting, fatigue, loss of appetite, mouth sores, low blood count, diarrhoea and peripheral neuropathy [85]. Apoptotic efficacy of oxaliplatin is enhanced by the addition of mangiferin, as mangiferin inhibits NFκB (see above) [13,14,48,77] and is thought to increase the sensitivity of malignant cells to apoptotic cell death [48].

Du Plessis-Stoman et al. demonstrated the positive effect of mangiferin on oxaliplatin action in HeLa cells and HT29 cells through use of IC_50_ assays [48]. When stained with tryptan blue, cells treated with oxaliplatin and mangiferin displayed fewer non-viable cells than those treated with oxaliplatin only, indicating that there was less necrosis, suggesting the apoptotic pathway for cell death was preferred [48]. Co-administration of mangiferin with oxaliplatin increased caspase 3 activation in HeLa and HT29 cell lines relative to cells that only received oxaliplatin, further implicating the apoptotic pathway of cell death was favoured, thus reducing inflammation [48].

Du Plessis-Stoman et al. have suggested that mangiferin only exhibits NFκB inhibition when used with platinum containing complexes, as they found that treatment of normal cells with mangiferin alone resulted in increased NFκB activity [48]. When treated with mangiferin and oxaliplatin, the level of NFκB inhibition was similar to cells treated with oxaliplatin alone. However, in the presence of mangiferin, the oxaliplatin IC_50_ was 3.4 times lower in the cells receiving both treatments [48]. In addition, when assessing changes in cell cycle, mangiferin caused a delay in S-phase only when used in conjunction with oxaliplatin [48]. On treatment with oxaliplatin or mangiferin alone, a G_2_/M phase cell cycle arrest was noted.

Both oxaliplatin and mangiferin are implicated in the mitochondrial pathway of apoptosis through reduction of mitochondrial membrane potential. However, cells treated with mangiferin and oxaliplatin did not show a significantly different mitochondrial membrane potential to those treated with oxaliplatin alone [48].

Evidence indicates that mangiferin increases the efficacy of oxaliplatin at inducing cell death in malignant cells.

#### 3.1.2. Etoposide

As discussed above (Section 2.5.1 on Nrf2), mangiferin protects against etoposide induced oxidative damage in human umbilical cord blood mononuclear cells by promoting Nrf2 signalling to activate a number of antioxidant enzymes [13]. Side effects such as myelo-suppression are also reduced. As discussed earlier, literature indicated that mangiferin causes G_2_/M phase cell cycle arrest. Etoposide targets cells in this phase [86]. In addition, oxidative damage in response to etoposide may result in p53 activation. However, when the effect of mangiferin on etoposide efficacy was studied, HL-60 cells were used, which lack wild type p53, thus further experimentation is required to elucidate this effect [13].

#### 3.1.3. Doxorubicin

Louisa et al. (2014) reported that mangiferin increased the efficacy of doxorubicin in MCF-7 [33]. Cells were initially incubated with a low concentration of doxorubicin for 10 days. The apoptotic rate was measured and found to be reduced, indicating the development of drug resistance. Thereafter, cells were treated with mangiferin and at high concentrations mangiferin significantly reduced cell viability through reduced expression of P-glycoprotein, which acts as a multidrug transporter. The efficacy of mangiferin increased in a concentration dependent manner [33]. In this study it was found that mRNA levels associated with multidrug resistance associated protein-1 and breast cancer resistance protein were unaffected by mangiferin, unlike P-glycoprotein [33].

## 4. Bioavailability and Delivery of Mangiferin

Extraction, quantification, solubility and bioavailability of polyphenols, including mangiferin, are of relevance to clinical success. Bioavailability is dependent on bioaccessibility (quantity of compound released from the food matrix), solubility in gastrointestinal fluids, cellular uptake, compound metabolism and efficiency of the circulatory system [87,88]. Like many other polyphenols, the optimal health benefits of mangiferin are not fully realised due to poor water solubility and oral bioavailability (1.2% in rats) [89].

Using HPLC-MS, Hou et al. evaluated the pharmacokinetics (PK) of mangiferin following oral administration (0.1 g. 0.3 g and 0.9 g) in healthy male volunteers [90]. The point of maximum plasma concentration (38.64 ng/mL^−1^) was at approximately 1 h, and was surprisingly low considering the dose of 0.9 g. This outcome supports other published findings such as those reported by [89,91] in rats. Maximal plasma concentrations, both quantity and time, were enhanced when mangiferin was orally administered to rats as a polyherbal formulation, rather than as mangiferin alone [92]. Similarly, Ma et al., in a rat model, found that permeability and plasma concentrations were improved following administration of a phospholipid complex containing mangiferin, relative to administration of mangiferin alone [93]. However, in addition to whole body PK, intratumoral PK, influenced by packing density of solid tumour cells and components of the extracellular matrix, is also important [94], and these challenges could be addressed by co-formulation and innovative delivery modes.

Bioavailability can be influenced by the properties of the food matrix (composition and structure) and hence the oral bioavailability of bioactive compounds, in this case, mangiferin could be improved if the major limiting factors were characterised [88] and modes of delivery designed accordingly. McClements et al. developed a new system for the classification of factors limiting oral bioavailability of nutraceuticals such that the design of food matrices can be optimised for each nutraceutical. The classification system is largely based on bioaccessibility (liberation, solubilisation and interactions), absorption (mucus layer, bilayer permeability and tight-, active- and efflux- transporters), and transformation (chemical degradation and metabolism) [88]. Such a system assists with determining an optimal food matrix design that will maximise oral bioavailability e.g., the encapsulation of a compound with low bilayer permeability or the addition of components that may protect a compound, that is sensitive to metabolism, against enzymes in the gut [88]. Many of these characteristics need to be assessed for mangiferin in order to improve oral bioavailability.

Encapsulation of compounds has improved PK properties in general, and is particularly suitable for compounds such as mangiferin, that are poorly water soluble [50]. Spray-drying formulations can impact on retention of mangiferin in the particle as demonstrated by the comparison of a pectin formulation versus a chitosan polysaccharide, with pectin being found to have a better retention of mangiferin in the particles than a chitosan formulation [50]. Numerous types of nanovehicles have been developed, and many polysaccharide-based nanovehicles have been used for the delivery of anti-cancer drugs, some of which may interact with membrane receptors. (See Caro and Pozo for an overview on the application of polysaccharides as nanovehicles in cancer therapy [95]). Specialised polysaccharide-based nanovehicles may be suitable for the delivery of mangiferin. It is clear that further work is required with respect to improving bioavailability and delivery methods of mangiferin from fruit or supplement to tumour site. The design of a “smart vehicle” for the delivery of mangiferin to the tumour cells, rather than healthy cells, and for avoidance or minimisation of a delivery gradient within the solid tumour, the “smart vehicle” will likely need to be unique to mangiferin and possibly to the cancer type.

## 5. Toxicity

In addition to bioavailability and delivery of bioactive compound to enhance health, it is critical to consider toxicity of the compound. Being a natural compound, mangiferin exhibits minimal toxicity [34] and is generally regarded as non-toxic [28]. Stem-bark extract from *Mangifera indica* has only shown toxicity in animals when injected intra-peritoneally and after acute exposure [12]. Mangiferin’s reported toxic dose in mice is 400 mg/kg [28,50]. In experiments involving blood peripheral lymphocytes and hepatocytes of rats, mangiferin did not induce cytotoxicity, genotoxicity or mutagenicity [12]. However, in a more recent study by Prado et al. [96], oral administration of mangiferin in rodents demonstrated low acute and sub-chronic toxicity. Nonetheless, it is still anticipated that there is a wide safety margin for this compound when taken orally [96]. Due to the polyphenolic structure of mangiferin, it is likely to undergo biotransformation in the liver, and for this reason it is suggested that further investigation into the safety of mangiferin metabolites may be required [23].

## 6. Conclusions

Evidence strongly supports the link between mangiferin treatment and modulation of many molecular pathways to prevent the development and progression of cancer. Mangiferin is primarily implicated in down-regulating inflammation, causing cell cycle arrest, reducing proliferation/metastasis, promoting apoptosis in malignant cells and protecting against oxidative stress and DNA damage. Perhaps the most promising anti-proliferative effect observed on treatment with mangiferin was that seen during in vivo experiments where mangiferin reduced tumour volume to a similar extent as treatment with cisplatin. Literature consistently shows that mangiferin enhances the efficacy of pro-apoptotic chemotherapeutic agents, with the most evidence supporting synergistic effects with oxaliplatin, etoposide and doxorubicin. This is of particular interest when we consider that mangiferin exhibits low toxicity and has a wide oral safety margin, unlike other compounds with similar activity. However, the bioavailability and delivery of mangiferin requires further research and development.

Ultimately, there is strong evidence, in a number of pathways, for a protective effect of mangiferin. However, in some cases, there may be variation in effect due to dosage, origin of extract or cell line used. Furthermore, low water solubility as well as low oral bioavailability are two factors that limit clinical use at present, and further research efforts targeting appropriate delivery systems are required in order to improve clinical efficacy. In addition, investigations into in vivo effects are required to determine the significance of these results to human health. Clinical trials in humans could substantially improve our understanding of the macroscopic effects of mangiferin. Additionally, further investigation into mangiferin aglycone, may uncover a more sustainable way of achieving greater efficiency than that observed with mangiferin alone.

## Figures and Tables

**Table 1:** Summary of proposed beneficial effects of co-administration of mangiferin alongside chemotherapeutic agents.

Chemotherapeutic Agent	Cell Line	Reference	Evidence
Oxaliplatin	HeLa, HT29, HL60	[48]	Reduction in oxaliplatin IC50 values; counteracts resistance to oxaliplatin.
Etoposide	HL60, U937	[11,13]	Reduces oxidative stress. Protects normal cells without reducing sensitivity of HL60 to etoposide [13]. Activity of the drug is enhanced by mangiferin [11].
Doxorubicin	MCF7, U937	[13,33]	At high concentrations mangiferin can inhibit P-glycoprotein expression and chemosensitise for doxorubicin therapy [33]. Activity of the drug is enhanced by mangiferin [11].
Paclitaxel	Triple negative breast cancer	[60,62]	IRAK1 overexpression confers a growth advantage [62]. Mangiferin may inhibit *IRAK1* activation [60,62].
Cisplatin	U937	[11]	Inhibits ROS production [8]. Activity of the drug is enhanced by mangiferin; Impedes NFκB activation; Enhanced cell death in the presence of TNF [11].
Vincristine	U937	[11]	Inhibits ROS production [8]. Activity of the drug is enhanced by mangiferin; Impedes NFκB activation; Enhanced cell death in the presence of TNF [11].
Adriamycin	U937	[11]	Inhibits ROS production [8]. Activity of the drug is enhanced by mangiferin; Impedes NFκB activation; Enhanced cell death in the presence of TNF [11].
AraC	U937	[11]	Inhibits ROS production [8]. Activity of the drug is enhanced by mangiferin; Impedes NFκB activation; Enhanced cell death in the presence of TNF [11].

## Abbreviations

The following abbreviations are used in this manuscript:

AML	acute myeloid leukaemia
ARE	antioxidant response element
ATM	Ataxia telangiectasia mutated protein
ATR	Ataxia Telangiectasia and Rad3-related protein
ATZ	aminotriazole
Bax	Bcl-2 associated X protein
bcl-2	B Cell Lymphoma-2
bcl-xL	B Cell Lymphoma-extra large
B(a)P	benzo(a)pyrene
Bid	BH3 interacting domain
BIRC7	Baculoviral IAP Repeat Containing 7
Chk1	Checkpoint kinase 1
CHk2	Checkpoint Kinase 2
CDK1	Cyclin-Dependent Kinase 1
COX	Cyclooxygenase-2
CXCR4	C-X-C Chemokine Receptor type-4
DPPH	2,2-diphenyl-1-picrylhydrazyl
EMT	Epithelial to Mesenchymal Transition
ERK	Extracellular signal-Regulated Kinase
FADD	Fas Associated Death Domain
GSH	glutathione S-transferase
HO-1	heme oxygenase
H_2_O_2_	hydrogen peroxide
IARC	International Agency for Research on Cancer
ICAM-1	Intercellular Adhesion Molecule-1
IκB	Inhibitor of κB
IKK-α	Inhibitor of NFκB Kinase subunit-α
IKK-β	Inhibitor of NFκB Kinase subunit-β
IL-1R	Interleukin-1 Receptors
IL-6	Interleukin-6
IL-8	Interleukin-8
IRAK1	Interleukin-1 Receptor Activated Kinase 1
IRAK4	Interleukin-1 Receptor Activated Kinase 4
KEAP-1	Kelch-like ECH-associated protein-1
LDC	Less Developed Countries
LPS	lipopolysaccharide
maf	musculoaponeurotic fibrosarcoma
MAPK	Mitogen Activated Protein Kinase
MDCs	More developed countries
MMP	matrix metalloproteinase
MPP^+^	1-methyl-4-phenylpyridine
MTT	3-(4,5-dimethyl-2-thiozolyl)-2,5-diphenyl-2H-tetrazolium bromide
Myd88	Myeloid Differentiation Primary Response Gene 88
NEMO	NFκB Essential Modulator
NFκB	Nuclear Factor κ-light-chain-enhancer of activated B cells
NIK	NCK Interacting Kinase
NQO1	NAD(P)H: quinine reductases
Nrf2	Nuclear factor erythroid 2-Related Factor 2
PDG	peptidoglycan
PK	pharmacokinetics
PMA	phorbol-12-myristate-13-acetate
PPARү	Peroxisome Proliferator-Activated Receptor ү
ROS	Reactive oxygen species
SEAP	Secreted Embryonic Alkaline Phosphatase
SOD	superoxide
TAB1	Transforming growth factor beta-activated kinase 1-binding protein 1
TAB2	Transforming growth factor beta-activated kinase 1-binding protein 2
TAK1	Transforming growth factor beta-activated kinase 1
TLRs	Toll-like receptors
TNF	Tumour Necrosis Factor
TNFR	Tumour Necrosis Factor Receptor
TRADD	TNFR with Tumour Necrosis Factor Receptor type-1-Associated Death Domain protein
TRAF2	Tumour Necrosis Factor Receptor-Associated Factor 2
TRAF6	Tumour necrosis factor Receptor-Associated Factor 6
VEGF	Vascular Endothelial Growth Factor
XIAP	X linked Inhibitor of Apoptosis Protein
